# Pharmacokinetic Interactions of Clinical Interest Between Direct Oral Anticoagulants and Antiepileptic Drugs

**DOI:** 10.3389/fneur.2018.01067

**Published:** 2018-12-07

**Authors:** Alessandro Galgani, Caterina Palleria, Luigi Francesco Iannone, Giovambattista De Sarro, Filippo Sean Giorgi, Marta Maschio, Emilio Russo

**Affiliations:** ^1^Neurology Unit, Azienda Ospedaliero Universitaria Pisana, Pisa, Italy; ^2^Department of Science of Health, University Magna Graecia of Catanzaro, Catanzaro, Italy; ^3^UOSD Neurology, Center for Tumor-related Epilepsy, Regina Elena National Cancer Institute, Rome, Italy

**Keywords:** DOACs, antiepileptics, interactions, CYP, P-gp, AEDs, dabigatran, rivaroxaban

## Abstract

Direct oral anticoagulants (DOACs), namely apixaban, dabigatran, edoxaban, and rivaroxaban are being increasingly prescribed among the general population, as they are considered to be associated to lower bleeding risk than classical anticoagulants, and do not require coagulation monitoring. Likewise, DOACs are increasingly concomitantly prescribed in patients with epilepsy taking, therefore, antiepileptic drugs (AEDs), above all among the elderly. As a result, potential interactions may cause an increased risk of DOAC-related bleeding or a reduced antithrombotic efficacy. The objective of the present review is to describe the pharmacokinetic interactions between AEDs and DOACs of clinical relevance. We observed that there are only few clinical reports in which such interactions have been described in patients. More data are available on the pharmacokinetics of both drugs classes which allow speculating on their potential interactions. Older AEDs, acting on cytochrome P450 isoenzymes, and especially on CYP3A4, such as phenobarbital, phenytoin, and carbamazepine are more likely to significantly reduce the anticoagulant effect of DOACs (especially rivaroxaban, apixaban, and edoxaban). Newer AEDs not affecting significantly CYP or P-gp, such as lamotrigine, or pregabalin are not likely to affect DOACs efficacy. Zonisamide and lacosamide, which do not affect significantly CYP activity *in vitro*, might have a quite safe profile, even though their effects on P-gp are not well-known, yet. Levetiracetam exerts only a potential effect on P-gp activity, and thus it might be safe, as well. In conclusion, there are only few case reports and limited evidence on interactions between DOACs and AEDs in patients. However, the overall evidence suggests that the interaction between these drug classes might be of high clinical relevance and therefore further studies in larger patients' cohorts are warranted for the future in order to better clarify their pharmacokinetic and define the most appropriate clinical behavior.

## Introduction

The *direct-acting oral anticoagulants* (DOACs), also known as *non-vitamin K oral anticoagulants* (NOACs), are five drugs acting on coagulation cascade, without the use of anti-thrombin as a mediator, subdivided in factor Xa inhibitors (apixaban, edoxaban, and rivaroxaban) and direct thrombin inhibitors (argatroban and dabigatran).

Their indication in the clinical practice is as anticoagulants for primary and secondary prevention of ischaemic stroke, in patients suffering from non-valvular atrial fibrillation (AF) (1), but also for prevention and treatment of pulmonary embolism and deep venous thrombosis (2).

Strokes and cerebrovascular diseases represent the main cause (30–40%) of symptomatic epilepsy among elderly (3) and most of these patients need a chronic treatment with antiepileptic drugs (AEDs). Therefore, it is not rare that some patients might undergo concomitant treatment AEDs-DOACs and this co-treatment could lead to pharmacological interactions with serious consequences for patient's health. In particular, AEDs causing a reduced absorption or an increase of DOAC metabolism can cause a reduced antithrombotic efficacy of these drugs; conversely, a reduced DOAC metabolism can increase significantly the risk of bleeding in these patients [(4); and see below].

The aspect of drug-drug interactions is particularly important in persons with epilepsy, since optimal seizure control is often achieved only after different treatment attempts or using AEDs polytherapy (5). Furthermore, convulsive seizures expose patients to potential traumatic injuries that can be more dangerous in patients under anticoagulant treatment. Consequently, the potential interactions between DOACs and AEDs represent a field of particular clinical interest.

Aim of this review is to provide an overview on interactions between DOACs and AEDs using clinical and pharmacokinetic data. We considered only DOACs that are currently marketed in EU countries: edoxaban, rivaroxaban, apixaban, and dabigatran.

## Methods

**Figure 1:**
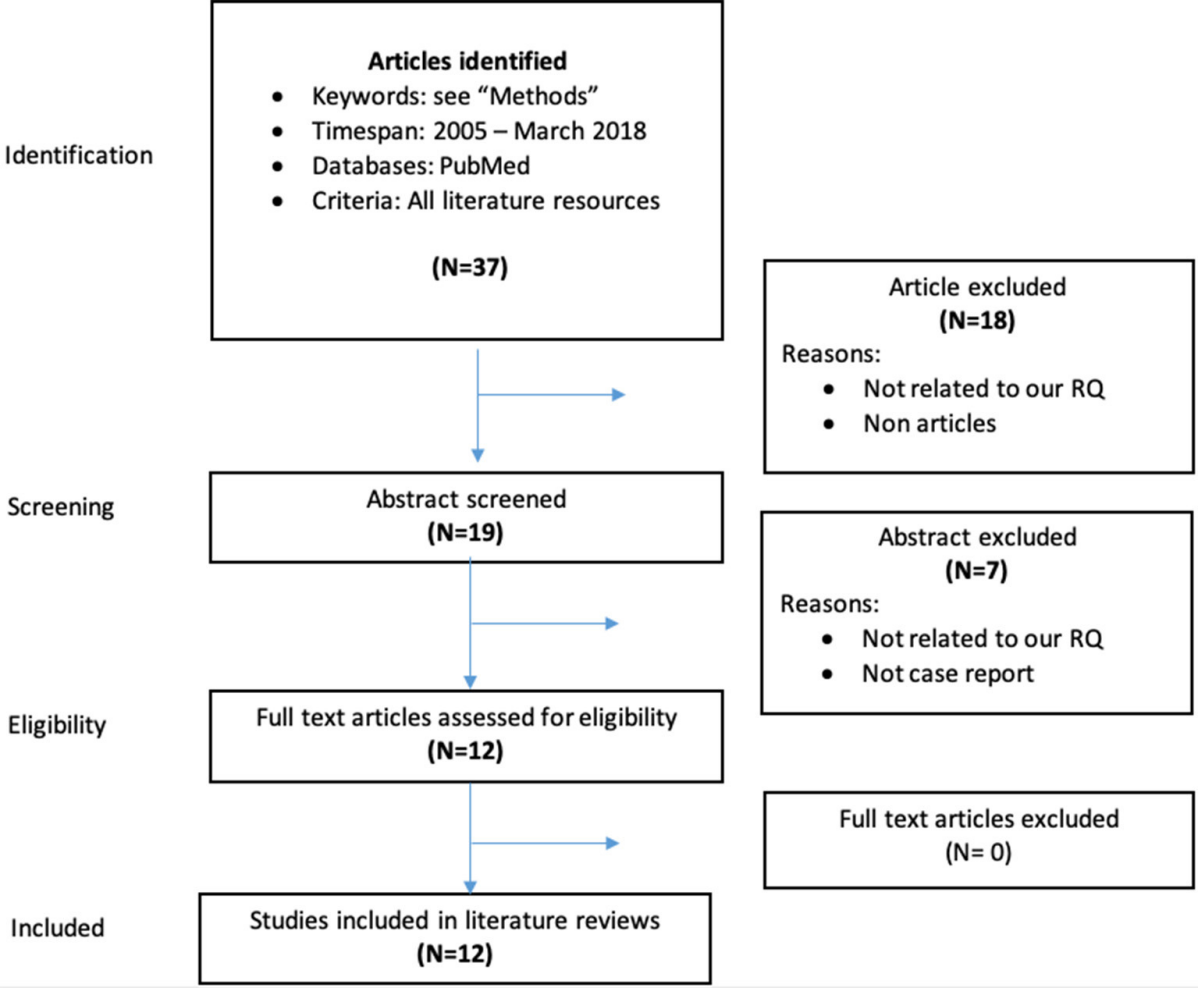
Data collection process for case reports and clinical series on interactions between DOACs and AEDs.

The articles on clinical series and case reports specifically addressing the interactions between DOACs and AEDs were selected starting from a PubMed search with the following search terms: “eslicarbazepine” or “felbamate” or “gabapentin” or “lamotrigine” or “levetiracetam” or “oxcarbazepine” or “perampanel” or “pregabalin” or “retigabine” or “rufinamide” or “stiripentol” or “tiagabine” or “topiramate” or “lacosamide” or “vigabatrin” or “zonisamide” or “phenobarbital” or “phenytoin” or “ethosuximide” or “carbamazepine” or “valproate,” and “dabigatran” or “rivaroxaban” or “apixaban” or “edoxaban,” with publication dates between 2005 and 2018. The Flow-Chart in Figure 1 details the process of inclusion/exclusion of the articles.

Data on pharmacokinetics of AEDs and DOACs for this review article were collected performing a search on PubMed using the following search terms: “eslicarbazepine,” “felbamate,” “gabapentin,” “lamotrigine,” “levetiracetam,” “oxcarbazepine,” “perampanel,” “pregabalin,” “rufinamide,” “stiripentol,” “tiagabine,” “topiramate,” “lacosamide,” “vigabatrin,” “zonisamide,” “phenobarbital,” “phenytoin,” “ethosuximide,” “carbamazepine,” or “valproate” and “CYP3A5” or “CYP2J2,” or “CYP3A4” or “P-gp,” or ”P-glycoprotein,” They were considered *in vitro* and *in vivo* experimental studies, and studies in humans from 1975 to March 2018. Similarly, it was subsequently performed another PubMed search, from 1999 to March 2018, for “dabigatran,” “rivaroxaban,” “apixaban,” “edoxaban” and “CYP3A5,” “CYP2J2,” “CYP3A4,” “P-gp,” “P-glycoprotein.” To reduce publication bias, we also searched the abstract proceedings of the international congresses by the International League Against Epilepsy (ILAE) and by the American Epilepsy Society.

The latter searches aimed at the definition of pharmacokinetic parameters and the most salient review papers, together with all product characteristics (SPCs) of the single drugs, were selected by the authors based on their experience in the field.

## Pharmacokinetic of DOACs

**Figure 2:**
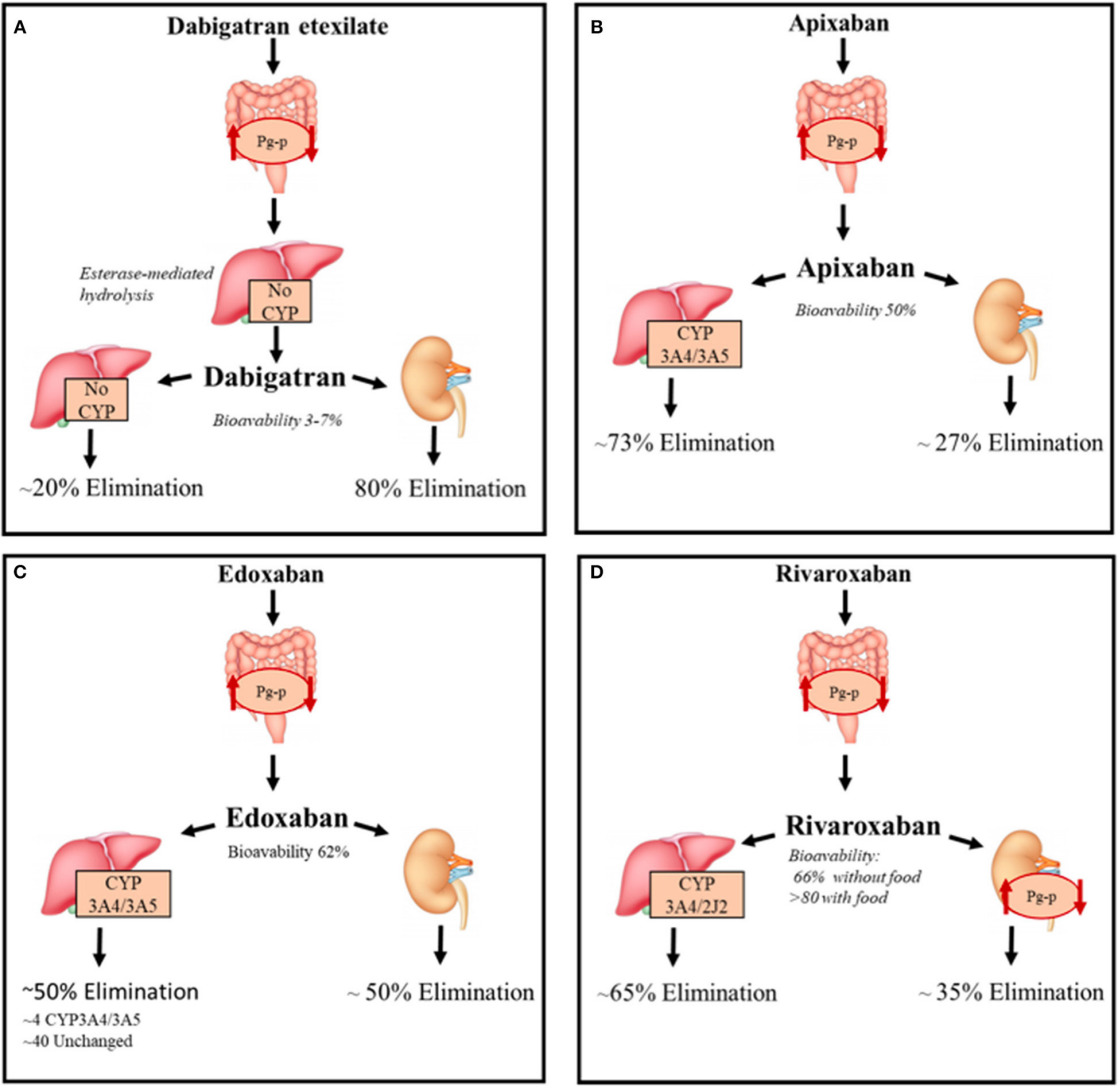
DOACs pharmacokinetic characteristics. Summary of the pharmacokinetic characteristics of DOACs with focus on the metabolization and elimination processes. **(A)** Direct thrombin inhibitor, **(B–D)** Direct factor Xa inhibitors. *CYP, Cytochromes P450; P-gp, P-glycoprotein 1*.

All DOACs pharmacokinetic features are summarized in Figure 2.

### Direct Thrombin Inhibitor

Dabigatran reversibly binds the active site of thrombin and it is administered as a pro-drug, dabigatran etexilate, since it is not absorbed by gastrointestinal tract after oral intake because of its high polarity; the etexilate form is rapidly hydrolyzed by carboxyl esterases (CES) to the active compound. The intestinal absorption of dabigatran etexilate, as well as other treated DOACs, depends on Permeability glycoprotein (*P-gp*) (6). The latter, is an ATP-dependent efflux transporter located in the plasma membrane of many different cell types; it regulates the absorption of xenobiotics from the gut lumen and is involved in the hepatic and renal excretion of these substances; it is also involved in blood-brain barrier permeability to drugs (7).

Bioavailability is 6.5% after administration, the lowest of all DOACs, is probably due to Pg-p intestinal excretion and low solubility of the pro-drug considering that it is not a substrate of cytochrome P-450 system. Considered a 12 h half-life, with a maximum concentration reduced by 30% after 4–6 h, dabigatran is administered twice a day (8).

This DOAC is dialyzable considering its very low binding with plasma proteins (~30%) and its 80% eliminated by kidneys (75% unchanged and 4% as active acyl-glucuronide metabolites), the remaining non-renal excretion is due to conjugation by uridine *diphosphate-glucuronyl-transferase* (UGT)2B15. Conjugation with activated glucuronic acid apparently represents the only metabolic modification of dabigatran (9). Food has no interaction with dabigatran, but the concurrent intake could decrease the plasma peak concentration (8).

### Direct Factor Xa Inhibitors

Apixaban, edoxaban and rivaroxaban are selective inhibitors of Xa factor (FXa) by binding its active site both when free or thrombin-bound.

Unlike dabigatran, these are not pro-drugs and have, when orally administered, an optimal and rapid absorption profile through the gastrointestinal tract that also depends on P-gp (9, 10) and this transporter also contributes to the renal excretion of rivaroxaban (11). The latter have a very high oral bioavailability (~90% with food), compared with apixaban and edoxaban (~ 50 and ~62% for apixaban and edoxaban, respectively).

Apixaban needs to be administered twice a day, whereas Edoxaban only once a day with a plasma half-life of 9–14 and 9–10 h, respectively, after administration of multiple doses. Rivaroxaban is also administered once a day due to a persistence of high concentration after 24 h from oral intake. FXa inhibitors are not dialyzable and plasma protein binding is higher for rivaroxaban and apixaban (~93%) compared to edoxaban (~55%) and are excreted unchanged for 27, 33, and 50% of their bioavailable dose, respectively (12–14).

These DOACs are substrates of the cytochrome P-450 system, and especially the CYP3A4 isoform (15, 16). In particular, rivaroxaban undergoes CYP3A4/3A5- and CYP2J2-mediated oxidative metabolism (18 and 14% of the total absorbed dose, respectively) (17). Apixaban is primarily metabolized by CYP3A4/3A5 and secondly by sulpho-transferase (SULT) 1A1, while edoxaban is minimally metabolized by CYP3A4/3A5 and mainly eliminated unchanged in bile (40%) (18).

None of the FXa inhibitors have interactions with food, have been tested in pregnancy and have shown any liver toxicity but dedicated safety studies should be realized to better define DOACs drug-induced liver injury (19).

## Pharmacokinetics of AEDs

The most important pharmacokinetic interactions of different AEDs with each other and with other classes of drugs, involve cytochrome P450 (CYP) and, to a lesser extent, the uridine diphosphate glucuronosyltransferase (UGT) system (20). Carbamazepine, phenytoin, and phenobarbital, among first-generation agents, are inducers of several enzymes such as CYP1A2, CYP2C9, CYP2C19, and CYP3A4, but also of UGTs and epoxide hydrolase (21–23).

Lamotrigine does not interfere significantly with drug metabolizing enzymes at low dosages, but at a dose higher than 300 mg/day, it has been proven to cause a reduction of 20% of levonorgestrel serum concentration (24). Valproate is able to inhibit the activity of CYP2C9, and, to a lesser extent CYP3A4 and CYP2C19, as well as UGT1A4 and UGT2B7 (25). By contrast, valproate does not inhibit CYP2D6, CYP1A2, and CYP2E1 (23, 26, 27).

Even though newer AEDs have a limited enzyme-inducing potential compared with older—generation compounds, some of them are involved in metabolic modifications. In particular, perampanel (at doses ≥8 mg/day), eslicarbazepine acetate, felbamate, oxcarbazepine (at doses ≥1,200 mg/ day), topiramate, levetiracetam and rufinamide (at doses ≥400 mg/day) bear weaker enzyme-inducing properties and may stimulate the activity of CYP3A4 and/or some UGT isoenzymes (21, 22, 28). Furthermore, oxcarbazepine, eslicarbazepine, felbamate, and topiramate show a weak inhibitory activity on CYP2C19 (29); stiripentol, on the other hand, is a strong inhibitor of CYP3A4, CYP2D6, CYP2C19, and CYP1A2 (30).

At therapeutic doses, zonisamide inhibits *in vitro* the activity of CYP2A6, CYP2C9, CYP2C19, and CYP2E1, but does not affect significantly CYP3A4, CYP1A2, and CYP2D6 (21). No data on the induction or inhibition capacity of ethosuximide, lacosamide, gabapentin, pregabalin, and vigabatrin, on human CYP or UGT isoenzymes have been published (21).

Some AEDs affect P-gp functions; in animal studies, levetiracetam, phenytoin and phenobarbital have been shown to cause P-gp induction, as well as carbamazepine for which there are also data in humans (31–33). An *in vitro* study shows that zonisamide is a weak inhibitor of P-gp with a CI50 of 267 μmol/L (34) All the main evidence on the effects of AEDs on P-gp and CYP3A4 (obtained from *in vivo* and *in vitro* studies) are listed in Table 1.

**Table 1:** Interactions between AEDs and P-gp or CYP3A4/3A5 and CYP2J2 systems.

**AEDs**	**P-gp**	**References**	**CYP3A4**	**CYP3A5/CYP2J2**	**References**
Eslicarbazepine acetate	Substrate (*in vitro*)	(35)	Weak inductor (*in vitro e vivo)*	NR	(21)
Felbamate	Substrate (*in vivo)*	(36)	Weak inductor/No effects (*in vitro*)	NR	(37, 26)
Gabapentin	Not substrate	(38)	NR	NR	(21)
Lamotrigine	No effects/substrate	(39)	No effects	No effects	(40)
Levetiracetam	Inductor/substrate *(in vivo)*	(41)	Weak inductor *(in vitro)*	No effects	(42)
Oxcarbazepine	NR		Inductor *(in vivo e vitro)*	Inductor 3A5 *(in vivo e vitro)*	(43)
Perampanel	No effects	(44)	Weak inductor *(in vitro)*	Weak inductor 3A5 *(in vitro)*	(28)
Pregabalin	No effects	(45)	No effects	No effects	(46)
Rufinamide	NR		Mild induction *(in vitro)*	No effects	(47)
Stiripentol	NR		Inhibitor *(in vitro)*	No effects	(48)
Tiagabine	NR		Substrate	No effects	(49)
Topiramate	No effects/substrate	(39)	Mild inductor *(in vitro)*	No effects	(50)
Lacosamide	No effects	(51)	No effects *(in vitro)*	No effects	(52)
Vigabatrin	NR		No effects	No effects	(21)
Zonisamide	Weak inhibitor	(34)	No effects/substrate	No effects	(53)
Phenobarbital	Inductor/substrate	(54)	Inductor	No effects	(55)
Phenytoin	Inductor/substrate *(in vivo)*	(56)	Inductor/substrate *(in vivo)*	NR	(55)
Ethosuximide	NR		Substrate	NR	(26)
Carbamazepine	Inductor *(in vivo)*	(57)	Substrate/inductor *(in vitro and vivo)*	NR	(58)
Valproate	Inductor/inhibitor *(in vitro)*	(59, 60)	Inductor/weak inhibitor *(in vitro)*	NR	(25, 61)

The main interaction between DOACs and AEDs are related to the effects of the two classes of drugs on CYP3A4 and vice-versa and can be hypothesized by knowing their effects on these targets; for P-gp, interactions are less intuitive.

## Case Reports

We performed a detailed search, including Pubmed publications and abstract proceedings of the international congresses by the International League Against Epilepsy (ILAE) and by the American Epilepsy Society, of all clinical descriptions, without language limits, concerning all the AEDs and all of the DOACs in the search. Unfortunately, up to date, there are only 12 clinically relevant articles available on this topic (Table 2).

**Table 2:** Clinical experiences on interaction between DOACs and AEDs.

**AEDs**	**Dabigatran**	**Rivaroxaban**	**Apixaban**
Eslicarbazepine acetate	/	/	/
Felbamate	/	/	/
Gabapentin	/	/	/
Lamotrigine	/	/	/
Levetiracetam	/	/	/
Oxcarbazepine	/	(62)	/
Perampanel	/	/	/
Pregabalin	/	/	/
Retigabin	/	/	/
Rufinamide	/	/	/
Stiripentol	/	/	/
Tiagabine	/	/	/
Topiramate	/	/	/
Lacosamide	/	/	/
Vigabatrin	/	/	/
Zonisamide	/	/	/
Phenobarbital	(63)	/	(64)
Phenytoin	(63) (66) (67) (68)	(65) (66)	(66)
Ethosuximide	/	/	/
Carbamazepine	(69)	(70) (71) (72)	/
Valproate	/	(73)	/

### Phenytoin

The AED for which most of studies and interactions with DOACs have been reported is phenytoin, which bears relevant clinical interactions with dabigatran, rivaroxaban, and apixaban. In 2017, Chang et al. found that in a large cohort of 91.330 patients suffering from non-valvular AF and treated with dabigatran, rivaroxaban or apixaban, there was a higher risk of major bleeding when the patients were taking phenytoin (N-7158) for concomitant epilepsy, as compared with patients not assuming this drug with an adjusted incidence rate difference (99% CI) per 1,000 person-years of 52.31 (32.18–72.44; *p* < 0.01). However, this study bears the strong limitations of a Health Insurance database system analysis (including the lack of detailed clinical/radiological information on the single patients) (66). In the same year, Hager et al. (68) described the occurrence of a left atrial thrombus in a 70-years-old patient whit a clinical history of hypertension, persistent AF, heterozygous factor V Leiden, recurrent deep venous thrombosis (DVT), and a pulmonary embolus, in co-treatment with atenolol, betahistine, diltiazem, valsartan, phenytoin 300 mg orally QD, and dabigatran etexilate 150 mg BID.

Phenytoin can affect both the absorption and metabolism of dabigatran, suggesting that could have led to a decreased anticoagulant effect and the development of atrial thrombus. Clinical relevance of this drug interaction has not been well described; anyway, the co-administration should be avoided (68). In 2016, Wiggins et al. had hypothesized the same type of interaction. They showed undetectable serum levels of dabigatran in a 45-years-old Afro-American male patient with AF treated also with phenytoin indicating that this drug could have a significant influence on dabigatran's metabolism and that this patient was at high risk for stroke (67).

Similarly, Becerra et al. (65) observed, in the first case documenting laboratory interaction between rivaroxaban and phenytoin, that DOAC levels were considerably low in a 48-years-old woman with cerebral vein thrombosis receiving also phenytoin, a combined CYP3A4 and P- glycoprotein inducer, which might reduce rivaroxaban levels (65).

### Phenobarbital

Phenobarbital may bear relevant interactions, too. In 2014, Chin et al. (63) evaluated median dose- corrected steady-state plasma dabigatran concentration (60 lg/L; range 9–279) in 52 patients (38–94 years). The dose-corrected concentration in a patient with co-administration of phenobarbital and dabigatran etixilate 110 mg BID was found 3 standard deviations below the cohort mean (concentration of 9 lg/L; dabigatran = 0.04l g/L per mg/day, z-score of the log-transformed dabigatran = −3.25). Authors hypothesized that this could occur via P-gp induction (63). In 2018, King et al. (64) reported the case of a 77-years-old patient on low-dose phenobarbital treatment for essential tremor, who was diagnosed with AF and, after dabigatran (150 mg BID) failure, she was switched to apixaban 5 mg BID.

In the following year, she suffered from two distinct episodes of cardioembolic stroke, and apixaban serum levels were lower (89 ng/mL approximately 11 h post-dose) than the expected therapeutic concentration. Furthermore, after phenobarbital discontinuation, the DOAC concentration rose to normal levels (361 ng/mL; unknown time post-dose) confirming a direct effect (64).

### Carbamazepine

Carbamazepine may affect the anticoagulant efficacy of dabigatran and rivaroxaban. In 2016, Laureano et al. described 2 patients, a 53-years-old man with epilepsy and AF (CHADS2 score of 2) and a 66-years-old woman with bipolar disorder, previous pulmonary embolism, and right leg deep vein thrombosis, receiving carbamazepine and dabigatran 150 mg BID. In both patients, dabigatran serum concentrations were reduced (steady state 24 ng/mL and 20 ng/mL, respectively), effect probably due to induction of P-gp by carbamazepine (69). In 2017, Stollberger and Finsterer, described the case of a 55-years-old Caucasian male, suffering from recurrent venous thrombosis in treatment with rivaroxaban (10 mg BID) and carbamazepine (900 mg/die) for structural epilepsy with complex partial seizures and secondary generalization. He was hospitalized because of increasing pain and swelling of his right leg starting spontaneously. Sonography showed a thrombosis of the right popliteal and femoral vein and analysis of drug concentrations showed a serum-carbamazepine level in the therapeutic range while anti-Xa activity was low (< 20 ng/ml) (70). Independently, in 2018, Burden et al. reported the case of a 71-years-old woman with clinical history of pulmonary embolism, subjected to the same therapy as the previous case. Presented to the Emergency department with acute onset shortness of breath, chest pain and palpitations, computed tomographic pulmonary angiography (CTPA) revealed multiple bilateral pulmonary emboli. Carbamazepine was hypothesized to be responsible of the DOAC inefficacy as the anti Xa activity was reduced in both cases (71).

Finally, Risselada et al. reported in 2013, only in Dutch language, a case of a 53-years-old man who underwent a partial knee arthroplasty and 4 days before developing a pulmonary embolism, whose symptoms started 1 day after he was switched from prophylactic dalteparin 5000 IE QD to rivaroxaban 10 mg one a day. Being the patient also in therapy with carbamazepine 600 mg BID for epilepsy, the authors of the case report hypothesized that pulmonary embolism was caused by a decrease in serum rivaroxaban levels due to the enzymatic induction of CYP3A4 by carbamazepine (72).

### Valproate and Oxcarbazepine

Rivaroxaban efficacy may also be affected by valproate and oxcarbazepine. In 2014, Stollberger and Finsterer described the case of an 88-years-old female patient, taking valproate and rivaroxaban 15 mg/die together, and whose anti-Xa activity was higher than expected. Indeed, coagulation tests after 28 h rivaroxaban-intake showed INR 2.26, PT 35%, aPTT 38.3 s and anti-Factor Xa-activity 2.00 U/m.

Even after the DOAC withdrawal, it took several days before coagulation was normalized, despite the short half-life of rivaroxaban (5–9 h) (16). The authors themselves acknowledged a potential key role of the patient's poor renal function and low body-mass-index (eGFR 34–42 ml/min/1.73 m^2^ and BMI = 19.95), but they could not exclude a drug interaction between rivaroxaban and valproate (73). The potential role of oxcarbazepine as a rivaroxaban inhibitor was suggested by Serra et al. (62), which described the case of a 68 years old man, suffering from permanent AF who had been put on rivaroxaban treatment, before undergoing external electrical cardioversion. He eventually did not undergo cardioversion because he developed a left atrial thrombosis despite DOAC treatment. The authors supposed that the thrombotic event was due to the interaction between rivaroxaban and oxcarbazepine, which the patient was taking for epilepsy, and which, as a strong CYP3A4 inducer, could have reduced rivaroxaban efficacy (62).

## Discussion and Conclusions

In this focused review, we summarized the clinical data available on the potential interactions existing between DOACs and AEDs. Although most of the clinical descriptions are merely anecdotal and do not allow further speculations, we can summarize that some old AEDs might modify DOACs efficacy; phenytoin, carbamazepine and phenobarbital might reduce significantly all DOACs efficacy while for oxcarbazepine and valproate, there are some data demonstrating a reduction of rivaroxaban efficacy, even though an interaction with other DOACs cannot be excluded. Finally, the interaction between phenobarbital and dabigatran has been better studied and it seems very convincing that phenobarbital reduces significantly dabigatran blood levels and efficacy. Based on their well-known enzymatic induction effects, phenytoin, carbamazepine and phenobarbital all potentially decrease the efficacy of rivaroxaban, apixaban, and edoxaban. One case report suggests that this would be the case also for dabigatran, even though this interaction was not easily predicted by knowing dabigatran CYP metabolism. This further emphasizes that specific predictions on the interactions between DOACs and AEDs are difficult, that many more clinical data are needed and that predictions based only on theoretical models might lead to wrong assumptions. However, theoretically based on the well-known effects of DOACs and AEDs on CYP and P-gp, several interactions can be hypothesized and should be kept in mind when starting a therapy with AEDs and DOACs.

Concerning newer AEDs, it can be speculated that those not affecting significantly CYP or Pg-p are not likely to affect DOACs efficacy and thus may be safer; this would be the case for lamotrigine or pregabalin. Lacosamide and zonisamide do not affect significantly CYP 3A4 activity, but their effects on Pg-p are not well-known yet. If the latter will be shown to be weak, they might be a good choice for patients on DOACs. For levetiracetam (which is otherwise considered quite neutral and “safe” in terms of pharmacokinetics interactions with many common drugs), an effect on CYP has not been shown but, since this AED may induce Pg-p activity, and is a substrate itself of this transporter, its safety in patients taking DOACs still needs to be demonstrated. Valproate and oxcarbazepine are AEDs still largely used in epileptic patients. Concerning valproate, its use is likely to affect significantly the pharmacokinetics of DOACs, as it affects significantly both CYP and Pg-p activity *in vitro*; the only case report available on valproate and rivaroxaban apparently contradicts this speculation, as it showed a reduced anti-Xa activity in 1 patient taking both drugs. However, the renal comorbidity of this patient probably played a role in these results. Oxcarbazepine, which is predicted to induce CYP activity, might also affect in a relevant way DOACs metabolism.

In conclusion, when a clinician has to choose an AED in a patient already taking DOACs, he might potentially choose among different second and third generation compounds which possess similar, significant, antiepileptic activity.

On other hand, it might be more complicated to start anticoagulants in patients with an established epilepsy that is well-controlled by old AEDs, and especially phenytoin, carbamazepine, phenobarbital or valproate. In these patients it might be risky, in terms of seizures recurrence, to modify an established AED. Such risk might be even increase when they are under anticoagulant drugs, i.e., at higher risk of major bleeding due to traumatic injury. Furthermore, previously pharmaco-resistant epileptic patients often take a combination of two or more of these AEDs at the same time, which further complicates predicting their effect on drug metabolism. In these patients, it might be still a reasonable clinical choice to use classical anticoagulants, such as warfarin, and tailoring its dosage in the single subject based on their INR values.

It is clear that population-based studies are needed to establish whether the pharmacological interactions between the two classes of drugs really represent a problem of clinical interest. Potentially, these aspects could be addressed at least in two ways. First, in order to establish the safest pharmacological combinations, it would be useful to perform cohort's studies comparing the compatibility between the most frequently administered AEDs and DOACs, starting at least with the ones which have the lower influence on CYP3A4, CYP3A5, and Pg-p (e.g., lamotrigine, pregabalin, lacosamide or levetiracetam). Another approach might consist in assessing *in vivo*, in the single patients taking both DOACs and AEDs, the efficacy of DOACs. Actually, one of the main reasons why DOACs have been developed is indeed to avoid the problem of strict coagulation monitoring, which is needed for patients using Vitamin-K antagonists. Some epileptic patients might represent one of the special populations in which such monitoring is indicated also for DOACs (which are considered, in any case safer, in terms of bleeding risk, than old anticoagulants, and thus to be preferred also in this population of subjects). Unfortunately, nowadays it is difficult to evaluate *in vivo* the antithrombotic effects of DOACs, and especially for those acting on factor Xa. These tests are not routinely available in some laboratories and their use needs a specific expertise. Moreover, mainly due to technical reasons, there are still differences in the results obtained among different laboratories and even among different specific DOACs within the same laboratory (74, 75). Hopefully, when in the future these methods (or different ones) will be more reproducible and approachable by different laboratories, it will be possible to assess in the single patient the existence of potential interactions between the DOAC and the AED(s) he/she is taking; once in the single patient such interaction has been excluded, it is likely that they would not need further evaluations (as usual for DOACs).

In conclusion, the risk of drug-drug interaction might be significant among patients taking AEDs and DOACs simultaneously, at least for some AEDs; future studies will help to better quantify this risk and to facilitate an optimal therapeutic handling of these patients.

## Limitations

The main limitations of this review consist in the lack of *in vivo/clinical* studies specifically addressing interactions between most DOACs and most AEDs, and the results reported are mainly speculative based on the knowledge of their pharmacokinetics features. Concerning some AEDs, we do not even know these effects in detail, and thus, their interaction with DOACs are not even predictable yet. The few data available from case reports are not strong enough to allow drawing definitive conclusions.

## Author Contributions

AG, CP, LI, GD, and FG: Text writing, bibliographic research and literature analysis. MM: Text review. ER: Text review and team coordination.

### Conflict of Interest Statement

ER has received speaker fees and participated to advisory boards for Eisai. MM has received speaker fees and participated to advisory boards for Eisai and UCB Pharma. The remaining authors declare that the research was conducted in the absence of any commercial or financial relationships that could be construed as a potential conflict of interest.

## References

[1] Kirchhof P Benussi S Kotecha D Ahlsson A Atar D Casadei B. 2016 ESC Guidelines for the management of atrial fibrillation developed in collaboration with EACTS. Eur Heart J. (2016) 37:2893–962. 10.1093/eurheartj/ehw210 27567408

[2] Ghazvinian R Gottsäter A Elf JL. Efficacy and safety of outpatient treatment with direct oral anticoagulation in pulmonary embolism. J Thrombosis Thrombolysis (2018) 45:319–24. 10.1007/s11239-017-1607-9 29305675 PMC5818558

[3] Wang JZ Vyas MV Saposnik G Burneo JG. Incidence and management of seizures after ischemic stroke. Neurology (2017) 89:1220–8. 10.1212/WNL.0000000000004407 28835405

[4] Stöllberger C Finsterer J. Concerns about the use of new oral anticoagulants for stroke prevention in elderly patients with atrial fibrillation. Drugs Aging (2013) 30:949–58. 10.1007/s40266-013-0119-3 24170233

[5] Devinsky O Vezzani A O'Brien TJ Jette N Scheffer IE de Curtis M Epilepsy. Nat Rev Dis Primers (2018) 4:18024 10.1038/nrdp.2018.24 29722352

[6] Stangier J Clemens A. Pharmacology, pharmacokinetics, and pharmacodynamics of dabigatran etexilate, an oral direct thrombin inhibitor. Clin Appl Thromb Hemost. (2009) 15(Suppl. 1):9S−16S. 10.1177/1076029609343004 19696042

[7] Wolking S Schaeffeler E Lerche H Schwab M Nies AT. Impact of genetic polymorphisms of ABCB1 (MDR1, P-Glycoprotein) on drug disposition and potential clinical implications: update of the literature. Clin Pharmacokinet. (2015) 54:709–35. 10.1007/s40262-015-0267-1 25860377

[8] Antonijevic NM Zivkovic ID Jovanovic LM Matic DM Kocica MJ Mrdovic IB. Dabigatran—metabolism, pharmacologic properties and drug interactions. Curr Drug Metab. (2017) 18:622–35. 10.2174/1389200218666170427113504 28460624

[9] Voukalis C Lip GY Shantsila E. Drug-drug interactions of non-vitamin K oral anticoagulants. Expert Opin Drug Metab Toxicol. (2016) 12:1445–61. 10.1080/17425255.2016.122503 27535163

[10] Härtter S Sennewald R Nehmiz G Reilly P. Oral bioavailability of dabigatran etexilate (Pradaxa®) after co-medication with verapamil in healthy subjects. Br J Clin Pharmacol. (2013) 75:1053–62. 10.1111/j.1365-2125.2012.04453.x 22946890 PMC3612723

[11] Gnoth MJ Buetehorn U Muenster U Schwarz T Sandmann S. *In vitro* and *in vivo* P-glycoprotein transport characteristics of rivaroxaban. J Pharmacol Exp Therapeut. (2011) 338:372–80. 10.1124/jpet.111.180240 21515813

[12] Kubisz P Stanciakova L Dobrotova M Samos M Mokan M Stasko J. Apixaban—Metabolism, pharmacologic properties and drug interactions. Curr Drug Metab. (2017) 18:609–21. 10.2174/1389200218666170424151551 28440204

[13] Kvasnicka T Malikova I Zenahlikova Z Kettnerova K Brzezkova R Zima T. Rivaroxaban—Metabolism, pharmacologic properties and drug interactions. Curr Drug Metab. (2017) 18:636–42. 10.2174/1389200218666170518165443 28524005

[14] Poulakos M Walker JN Baig U David T. Edoxaban: a direct oral anticoagulant. Am J Heal Pharm. (2017) 74:117–29. 10.2146/ajhp150821 28122753

[15] Perzborn E Roehrig S Straub A Kubitza D Misselwitz F. Rivaroxaban: a new oral factor Xa inhibitor. Arteriosclerosis Thrombosis Vascular Biol. (2010) 30:376–81. 10.1161/ATVBAHA.110.202978 20139357

[16] Mueck W Schwers S Stampfuss J. Rivaroxaban and other novel oral anticoagulants: pharmacokinetics in healthy subjects, specific patient populations and relevance of coagulation monitoring. Thrombosis J. (2013) 11:10. 10.1186/1477-9560-11-10 23809871 PMC3726366

[17] Weinz C Schwarz T Kubitza D Mueck W Lang D. Metabolism and excretion of rivaroxaban, an oral, direct factor xa inhibitor, in rats, dogs, and humans. Drug Metab Disposit. (2009) 37:1056–64. 10.1124/dmd.108.025569 19196845

[18] Bathala MS Masumoto H Oguma T He L Lowrie C Mendell J. Pharmacokinetics, biotransformation, and mass balance of edoxaban, a selective, direct factor Xa inhibitor, in humans. Drug Metab Disposit. (2012) 40:2250–5. 10.1124/dmd.112.046888 22936313

[19] Ingrasciotta Y Crisafulli S Pizzimenti V Marcianò I Mancuso A Andò G. Pharmacokinetics of new oral anticoagulants: implications for use in routine care. Expert Opin Drug Metab Toxicol. (2018) 14:1057–69. 10.1080/17425255.2018.1530213 30277082

[20] Zaccara G Perucca E. Interactions between antiepileptic drugs, and between antiepileptic drugs and other drugs. Epileptic Disord. (2014) 16:409–31. 10.1684/epd.2014.0714 25515681

[21] Patsalos PN Drug interactions with the newer antiepileptic drugs (AEDs)—Part 1: pharmacokinetic and pharmacodynamics interactions between AEDs. Clin Pharmacokinet. (2013) 52:927–66. 10.1007/s40262-013-0087-0 23784470

[22] Patsalos PN Drug interactions with the newer antiepileptic drugs (AEDs)—Part 2: pharmacokinetic and pharmacodynamics interactions between AEDs and drugs used to treat non-epilepsy disorders. Clin Pharmacokinet. (2013) 52:1045–61. 10.1007/s40262-013-0088-z 23794036

[23] Perucca E. Clinically relevant drug interactions with antiepileptic drugs. Br J Clin Pharmacol. (2006) 61:246–55. 10.1111/j.1365-2125.2005.02529.x 16487217 PMC1885026

[24] Sidhu J Job S Singh S Philipson R. The pharmacokinetic and pharmacodynamic consequences of the co-administration of lamotrigine and a combined oral contraceptive in healthy female subjects. Br J Clin Pharmacol. (2006) 61:191–9. 10.1111/j.1365-2125.2005.02539.x 16433873 PMC1885007

[25] Zhou SF Xue CC Yu XQ Li C Wang G Clinically important drug interactions potentially involving mechanism-based inhibition of cytochrome P4503A4 and the role of therapeutic drug monitoring. Ther Drug Monit. (2007) 29:687–710. 10.1097/FTD.0b013e31815c16f5 18043468

[26] Patsalos PN Perucca E. Clinically important drug interactions in epilepsy: general features and interactions between antiepileptic drugs. Lancet Neurol. (2003) 2:347–56. 10.1016/S1474-4422(03)00409-5 12849151

[27] Patsalos PN Perucca E. Clinically important drug interactions in epilepsy: interactions between antiepileptic drugs and other drugs. Lancet Neurol. (2003) 2:473–81. 10.1016/S1474-4422(03)00483-6 12878435

[28] Patsalos PN. The clinical pharmacology profile of the new antiepileptic drug perampanel: a novel noncompetitive AMPA receptor antagonist. Epilepsia (2015) 56:12–27. 10.1111/epi.12865 25495693

[29] Patsalos PN Fröscher W Pisani F van Rijn CM. The importance of drug interactions in epilepsy therapy. Epilepsia (2002) 43:365–85 10.1046/j.1528-1157.2002.13001.x 11952767

[30] Johannessen Landmark C Patsalos PN Drug interactions involving the new second- and third-generation antiepileptic drugs. Expert Rev Neurother. (2010) 10:119–40. 10.1586/ern.09.136 20021326

[31] Zhang C Kwan P Zuo Z Baum L. The transport of antiepileptic drugs by P-glycoprotein. Adv Drug Deliv Rev. (2012) 64:930–42. 10.1016/j.addr.2011.12.003 22197850

[32] O'Brien FE Dinan TG Griffin BT Cryan JF. Interactions between antidepressants and P-glycoprotein at the blood-brain barrier: clinical significance of *in vitro* and *in vivo* findings. Br J Pharmacol. (2012) 165:289–312. 10.1111/j.1476-5381.2011.01557.x 21718296 PMC3268186

[33] Akamine Y Yasui-Furukori N Ieiri I Uno T. Psychotropic drug-drug interactions involving P-glycoprotein. CNS Drugs (2012) 26:959–73. 10.1007/s40263-012-0008-z 23023659

[34] European Medicines Agency Zonisamide. Summary of Product Characteristics (2017). Available online at: https://www.ema.europa.eu/documents/product-information/zonegran-epar-product-information_en.pdf

[35] Zhang C Zuo Z Kwan P Baum L. *In vitro* transport profile of carbamazepine, oxcarbazepine, eslicarbazepine acetate, and their active metabolites by human P-glycoprotein. Epilepsia (2011) 52:1894–904. 10.1111/j.1528-1167.2011.03140.x 21692796

[36] Potschka H Fedrowitz M Löscher W. P-Glycoprotein-mediated efflux of phenobarbital, lamotrigine, and felbamate at the blood-brain barrier: evidence from microdialysis experiments in rats. Neurosci Lett. (2002) 327:173–6. 10.1016/S0304-3940(02)00423-8 12113905

[37] Italiano D Spina E de Leon J. Pharmacokinetic and pharmacodynamic interactions between antiepileptics and antidepressants. Expert Opin Drug Metab Toxicol. (2014) 10:1457–89. 10.1517/17425255.2014.956081 25196459

[38] Nakanishi H Yonezawa A Matsubara K Yano I. Impact of P-glycoprotein and breast cancer resistance protein on the brain distribution of antiepileptic drugs in knockout mouse models. Eur J Pharmacol. (2013) 710:20–8 10.1016/j.ejphar.2013.03.049 23588114

[39] Wang-Tilz Y Tilz C Wang B Tilz GP Stefan H. Influence of lamotrigine and topiramate on MDR1 expression in difficult-to-treat temporal lobe epilepsy. Epilepsia (2006) 47:233–9. 10.1111/j.1528-1167.2006.00414.X 16499746

[40] Yasam VR Jakki SL Senthil V Eswaramoorthy M Shanmuganathan S Arjunan K. A pharmacological overview of lamotrigine for the treatment of epilepsy. Expert Rev Clin Pharmacol. (2016) 9:1533–46. 10.1080/17512433.2016.1254041 27825017

[41] Moerman L Wyffels L Slaets D Raedt R Boon P De Vos F. Antiepileptic drugs modulate P-glycoproteins in the brain: a mice study with11C-desmethylloperamide. Epilepsy Res. (2011) 94:18–25. 10.1016/j.eplepsyres.2010.12.013 21277169

[42] Wright C Downing J Mungall D Khan O Williams A Fonkem E. Clinical pharmacology and pharmacokinetics of levetiracetam. Front Neurol. (2013) 4:192. 10.3389/fneur.2013.00192 24363651 PMC3850169

[43] May TW Korn-Merker E Rambeck B. Clinical pharmacokinetics of oxcarbazepine. Clin Pharmacokinet. (2003) 42:1023–42. 10.2165/00003088-200342120-0000 12959634

[44] Majid O Laurenza A Ferry J Hussein Z. Impact of perampanel on pharmacokinetics of concomitant antiepileptics in patients with partial-onset seizures: pooled analysis of clinical trials. Br J Clin Pharmacol. (2016) 82:422–30. 10.1111/bcp.12951 27038098 PMC4972158

[45] Schulze-Bonhage A. Pharmacokinetic and pharmacodynamic profile of pregabalin and its role in the treatment of epilepsy. Expert Opin Drug Metab Toxicol. (2013) 9:105–15. 10.1517/17425255.2013.749239 23205518

[46] Ben-Menachem E. Pregabalin pharmacology and its relevance to clinical practice. Epilepsia (2004) 45:13–8. 10.1111/j.0013-9580.2004.455003.x 15315511

[47] Perucca E Cloyd J Critchley D Fuseau E. Rufinamide: clinical pharmacokinetics and concentrationresponse relationships in patients with epilepsy. Epilepsia (2008) 49:1123–41. 10.1111/j.1528-1167.2008.01665.x 18503564

[48] Tran A Rey E Pons G Rousseau M d'Athis P Olive G. Influence of stiripentol on cytochrome P450-mediated metabolic pathways in humans: *in vitro* and *in vivo* comparison and calculation of *in vivo* inhibition constants. Clin Pharmacol Ther. (1997) 62:490–504. 10.1016/S0009-9236(97)90044-8 9390105

[49] Preissner S Kroll K Dunkel M Senger C Goldsobel G Kuzman D. SuperCYP: a comprehensive database on Cytochrome P450 enzymes including a tool for analysis of CYP-drug interactions. Nucleic Acids Res. (2010) 38:D237–43. 10.1093/nar/gkp970 19934256 PMC2808967

[50] Bialer M Doose DR Murthy B Curtin C Wang S-S Twyman RE. Pharmacokinetic interactions of topiramate. Clin Pharmacokinet. (2004) 43:763–80. 10.2165/00003088-200443120-00001 15355124

[51] European Medicines Agency Lacosamide. Summary of Product Characteristics (2013). Available online at: https://www.ema.europa.eu/documents/product-information/vimpat-epar-product-information_en.pdf

[52] Cawello W. Clinical pharmacokinetic and pharmacodynamic profile of lacosamide. Clin Pharmacokinet. (2015) 54:901–14. 10.1007/s40262-015-0276-0 25957198

[53] Leppik IE. Zonisamide: chemistry, mechanism of action, and pharmacokinetics. Seizure (2004) 13:5–9. 10.1016/j.seizure.2004.04.016 15511691

[54] Jing X Liu X Wen T Xie S Yao D Liu X. Combined effects of epileptic seizure and phenobarbital induced overexpression of P-glycoprotein in brain of chemically kindled rats. Br J Pharmacol. (2010) 159:1511–22. 10.1111/j.1476-5381.2009.00634.x 20233212 PMC2850407

[55] Spina E Pisani F De Leon J. Clinically significant pharmacokinetic drug interactions of antiepileptic drugs with new antidepressants and new antipsychotics. Pharmacol Res. (2016) 106:72–86. 10.1016/j.phrs.2016.02.014 26896788

[56] Alvariza S Fagiolino P Vázquez M Feria-Romero I Orozco-Suárez S. Chronic administration of phenytoin induces efflux transporter overexpression in rats. Pharmacol Rep. (2014) 66:946–51. 10.2165/11539230-000000000-00000 25443719

[57] Giessmann T May K Modess C Wegner D Hecker U Zschiesche M. Carbamazepine regulates intestinal P-glycoprotein and multidrug resistance protein MRP2 and influences disposition of talinolol in humans. Clin Pharmacol Ther. (2004) 76:192–200. 10.1016/j.clpt.2004.04.011 15371980

[58] Sugiyama I Murayama N Kuroki A Kota J Iwano S Yamazaki H. Evaluation of cytochrome P450 inductions by anti-epileptic drug oxcarbazepine, 10-hydroxyoxcarbazepine, and carbamazepine using human hepatocytes and HepaRG cells. Xenobiotica (2016) 46:765–74. 10.3109/00498254.2015.1118774 26711482

[59] Tang R Faussat AM Majdak P Perrot JY Chaoui D Legrand O. Valproic acid inhibits proliferation and induces apoptosis in acute myeloid leukemia cells expressing P-gp and MRP1. Leukemia (2004) 18:1246–51. 10.1038/sj.leu.2403390 15116123

[60] Eyal S Lamb JG Smith-Yockman M Yagen B Fibach E Altschuler Y. The antiepileptic and anticancer agent, valproic acid, induces P-glycoprotein in human tumour cell lines and in rat liver. Br J Pharmacol. (2006) 149:250–60. 10.1038/sj.bjp.0706830 16894351 PMC2014277

[61] Cerveny L Svecova L Anzenbacherova E Vrzal R Staud F Dvorak Z. Valproic acid induces CYP3A4 and MDR1 gene expression by activation of constitutive androstane receptor and pregnane X receptor pathways. Drug Metab Dispos. (2007) 35:1032–41. 10.1124/dmd.106.014456 17392393

[62] Serra W Li Calzi M Coruzzi P. Left atrial appendage thrombosis during therapy with rivaroxaban in elective cardioversion for permanent atrial fibrillation. Clin Pract. (2015) 5:788. 10.4081/cp.2015.788 26664717 PMC4653753

[63] Chin PKL Wright DFB Zhang M Wallace MC Roberts RL Patterson DM. Correlation between trough plasma dabigatran concentrations and estimates of glomerular filtration rate based on creatinine and cystatin C. Drugs R D (2014) 14:113–23. 10.1007/s40268-014-0045-9 24797400 PMC4070467

[64] King PK Stump TA Walkama AM Ash BM Bowling SM. Management of phenobarbital and apixaban interaction in recurrent cardioembolic stroke. Ann Pharmacother. (2018) 52:605–6. 10.1177/1060028018759938 29457494

[65] Becerra AF Amuchastegui T Tabares AH. Decreased rivaroxaban levels in a patient with cerebral vein thrombosis receiving phenytoin. Case Rep Hematol. (2017) 2017:4760612. 10.1155/2017/4760612 28875044 PMC5569874

[66] Chang S Chou IJ Yeh YH Chiou MJ Wen MS Kuo CT. Association between use of non-vitamin K oral anticoagulants with and without concurrent medications and risk of major bleeding in nonvalvular atrial fibrillation. Am Med Assoc. (2017) 318:1250–9. 10.1001/jama.2017.13883 28973247 PMC5818856

[67] Wiggins BS Northup A Johnson D Senfield J. Reduced anticoagulant effect of dabigatran in a patient receiving concomitant phenytoin. Pharmacother Publ. (2016) 36:e5–7. 10.1002/phar.1698 26846610

[68] Hager N Bolt J Albers L Wojcik W Duffy P Semchuk W. Development of left atrial thrombus after coadministration of dabigatran etexilate and phenytoin. Can J Cardiol. (2017) 33:554.e13–4. 10.1016/j.cjca.2016.10.022 28063739

[69] Laureano M Crowther M Eikelboom J Boonyawat K. Measurement of dabigatran drug levels to manage patients taking interacting drugs: a case report. Am J Med. (2016) 129:e247–8. 10.1016/j.amjmed.2016.06.017 27401948

[70] Stöllberger C Finsterer J. Recurrent venous thrombosis under rivaroxaban and carbamazepine for symptomatic epilepsy. Neurol Neurochir Pol. (2017) 51:194–6. 10.1016/j.pjnns.2017.01.010 28215696

[71] Burden T Thompson C Bonanos E Medford AR. Pulmonary embolism in a patient on rivaroxaban and concurrent carbamazepine. Clin Med. (2018) 18:103–5. 10.7861/clinmedicine.18-1-103 29436450 PMC6330908

[72] Risselada AJ Visser MJ van Roon EN. Pulmonary embolism due to interaction between rivaroxaban and carbamazepine. Ned Tijdschr Geneeskd. (2013) 157:A6568. 24382036

[73] Stollberger C Finsterer J. Prolonged anticoagulant activity of rivaroxaban in a polymorbid elderly female with non-convulsive epileptic state. Heart Lung (2014) 43:262–3. 10.1016/j.hrtlng.2014.03.004 24794785

[74] Salmonson T Dogné JM Janssen H Garcia Burgos J Blake P. Non-vitamin-K oral anticoagulants and laboratory testing: now and in the future: views from a workshop at the European Medicines Agency (EMA). Eur Heart J Cardiovasc Pharmacother. (2017) 3:42–7. 10.1093/ehjcvp/pvw032 28025213 PMC5216197

[75] Lippi G Favaloro EJ. Laboratory monitoring of direct oral anticoagulants (DOACs)-The perfect storm? Ann Transl Med. (2017) 5:6. 10.21037/atm.2017.01.03 28164091 PMC5253288

