# Corrigendum: Pharmacokinetic Interactions of Clinical Interest Between Direct Oral Anticoagulants and Antiepileptic Drugs

**DOI:** 10.3389/fneur.2019.01381

**Published:** 2020-01-29

**Authors:** Alessandro Galgani, Caterina Palleria, Luigi Francesco Iannone, Giovambattista De Sarro, Filippo Sean Giorgi, Marta Maschio, Emilio Russo

**Affiliations:** ^1^Neurology Unit, Azienda Ospedaliero Universitaria Pisana, Pisa, Italy; ^2^Department of Science of Health, University Magna Graecia of Catanzaro, Catanzaro, Italy; ^3^UOSD Neurology, Center for Tumor-Related Epilepsy, Regina Elena National Cancer Institute, Rome, Italy

**Keywords:** DOACs, antiepileptics, interactions, CYP, P-gp, AEDs, dabigatran, rivaroxaban

In the original article, there was an error. The manuscript states that “Apixaban and edoxaban need to be administered twice a day” whereas Edoxaban needs to be administered only once a day as stated in the European Product Information and Summary of Product Characteristics.

A correction has been made to the section **PHARMACOKINETIC OF DOACS**, subsection **Direct Factor Xa Inhibitors**:

“Apixaban, edoxaban and rivaroxaban are selective inhibitors of Xa factor (FXa) by binding its active site both when free or thrombin-bound.

Unlike dabigatran, these are not pro-drugs and have, when orally administered, an optimal and rapid absorption profile through the gastrointestinal tract that also depends on P-gp (9, 10) and this transporter also contributes to the renal excretion of rivaroxaban (11). The latter have a very high oral bioavailability (~90% with food), compared with apixaban and edoxaban (~50 and ~62% for apixaban and edoxaban, respectively).”

“Apixaban needs to be administered twice a day, whereas Edoxaban only once a day with a plasma half-life of 9–14 and 9–10 h, respectively, after administration of multiple doses. Rivaroxaban is also administered once a day due to a persistence of high concentration after 24 h from oral intake. FXa inhibitors are not dialyzable and plasma protein binding is higher for rivaroxaban and apixaban (~93%) compared to edoxaban (~55%) and are excreted unchanged for 27, 33, and 50% of their bioavailable dose, respectively (12–14).

These DOACs are substrates of the cytochrome P-450 system, and especially the CYP3A4 isoform (15, 16). In particular, rivaroxaban undergoes CYP3A4/3A5- and CYP2J2-mediated oxidative metabolism (18 and 14% of the total absorbed dose, respectively) (17). Apixaban is primarily metabolized by CYP3A4/3A5 and secondly by sulpho-transferase (SULT) 1A1, while edoxaban is minimally metabolized by CYP3A4/3A5 and mainly eliminated unchanged in bile (40%) (18).

None of the FXa inhibitors have interactions with food, have been tested in pregnancy and have shown any liver toxicity but dedicated safety studies should be realized to better define DOACs drug-induced liver injury (19).”

The authors apologize for this error and state that this does not change the scientific conclusions of the article in any way. The original article has been updated.

